# The Chelsea Workhouse Infirmary

**Published:** 1899-08-12

**Authors:** 


### THE CHELSEA WORKHOUSE INFIRMARY.

A very unfortunate difference has arisen between the matron and one of the medical officers at the Chelsea Workhouse Infirmary. It appears that in the early part of the summer a man was admitted suffering from umbilical hernia and bronchitis. For the relief of the hernia an operation was done by Dr. Horne, one of the assistant medical officers. At the end of about twelve days the sutures were removed, but the wound had not entirely healed, there still being some discharge from it. The next day, on the matron going her round, the patient complained of pain in his back, and to relieve this, and partly also, as she states, to lessen the likelihood of bed-sores occurring, she placed a soft pillow under his back. On finding that this had been done the medical officer remonstrated with the matron on what he considered an interference, and unfortunately the next time the wound was dressed it was found that it had become partially reopened. On this being discovered various somewhat angry letters passed, which to our mind seem to hint that even before this event the relations between the medical officers and the matron had not been characterised by that friendly co-operation in good works which is always desirable between responsible officers. At all events, in the end the whole matter came before the Board of Guardians, which being, like all such Boards, divided into more or less hostile camps, found in this accident an admirable opportunity for having a little squabble. About the surroundings of the affair, the assertions made on this side or that as to attempts to "hush the matter up," and all the rest of the hubbub, we have no concern. The real point is this, Was anything done contrary to orders? In regard to this we have the positive assurance of the matron that in all she did she merely acted as a nurse, using ordinary nursing appliances for the relief of the patient, and that not only did she not act act contrary to the orders given by the medical officer, but she did nothing opposed even to the spirit of those orders. She assures us that the orders given were not that the patient was "not to be moved," but that he was "not to be turned on to his side," and she points out the improbability of any medical man of experience giving an order that a man should "not be moved" for nearly a fortnight in the literal sense of the words, unless the necessary arrangements had been made at the same time for the prevention of bed-sores and other discomforts. She also states that, as a fact, the patient had been moved regularly for purposes of nature, and had had his back washed every day, raising himself from the bed for the purpose; and that, although she had not been present at the time, she had every reason to believe that all this had been done with the cognisance and consent of the medical officer who was attending on the case. Any movement caused by the insertion of the pillow, which has been made so much of, was, she says, quite within the limits of the movements which were daily being made. We are glad to have had this assurance, because the rule cannot be too strictly maintained that the duty of the nurse is to faithfully carry out the intention of the medical man. It is a serious matter that it should have gone forth, on the strength of paragraphs in papers which were apparently illinformed as to the facts, that a matron, who in such matters should always set an example to those under her, had been placing herself in opposition to the medical officer in the treatment of a case. Such statements cannot be too promptly denied. We print below a copy of a letter addressed to the Chelsea Board of Guardians by the matron in regard to the allegations made against her.

The subject came before the Board again last Wednesday, when a proposal to refer the matter to the General Committee for further inquiry was negatived by a majority of six to four.

Chelsea Infirmary, Cale Street, S.W.,
August 7th, 1899.

Ladies and Gentlemen,—My attention has been drawn to a report in the *West London Press* of some remarks made by Mr. Jeffery at the last meeting of the Board in connection with the patient Thomas Morgan.

These remarks are further accentuated in a letter addressed by Miss Grove to a certain nursing periodical, and are so misleading and inaccurate that I feel a reply is necessary.

Into the medical aspects of the case it is neither my intention nor my province to enter. The allegations are of a nature to carry with them their own refutation. Mr. Jeffery's assertion that I "ordered the patient to be moved" in spite of the doctor's orders is contrary to facts. The soft pillow which, in response to the patient's complaint to me of "soreness," I placed under his back to prevent the formation of a bedsore, was to avoid the necessity of moving him. The assistant medical officer's instructions, as I understood them from the nurse, were that the patient had not to be turned on his side, and my endeavour to ease his position in the manner described was in deference to those instructions. I deny that there was any intention or desire on my part to interfere with "medical treatment." I regarded the matter entirely from a nursing standpoint, honestly endeavouring to do my best for the patient. Thirteen days had elapsed since the performance of the operation. The last stitch had been removed the previous day. The man was reported as convalescent, except for the bronchitis from which he suffered. He was taking ordinary diet, and was even permitted to indulge in the luxury of a pipe. Under such circumstances it never occurred to me that the placing of a feather pillow under his back could be construed into "interference" with medical treatment. The depth of the pillow when evenly pressed down does not exceed one inch in thickness, consequently the alleged "strain," which had a duration of five minutes, must have been considerably less than would be produced of necessity night and morning when the patient's back was washed, against the doing of which no orders had been given.

With regard to the following statements, which I quote verbatim:—

(1) "As a consequence the wound reopened and serious hæmorrhage resulted." It is untrue; there was no hæmorrhage, and there is no proof whatever that the insertion of the pillow caused the wound to give way.

(2) "Dr. Horn remonstrated with the Matron, and she partially admitted the truth of his statements." It is likewise untrue; I never admitted that I interfered with the treatment. I acknowledged placing the pillow, but only in the ordinary course of nursing.

(3) "She made an understanding impossible by retorting that the doctor had neglected to use bandages on the wound, and therefore he was responsible." It is a mis-statement. What I maintained was this—and I am open to correction—that as the wound (from which there had been continuous slight oozing) was not strapped or otherwise supported after the removal of the last stitch, except by a bandage, it is unwarrantable to assume, in face of the violent cough to which the patient is subject, that the reopening of a portion of the wound was due to the pillow. In fact, the patient told me that he felt something give way while he was coughing, very shortly after the stitches were removed the day before.

I may add that there is no desire on my part to "hush up" the matter, for the very good reason that there is nothing to hush up. On the contrary, I should welcome the fullest investigation into the whole affair.—I beg to remain, ladies and gentlemen, yours obediently,

(Signed) Josephine L. de Pledge.

